# Early versus Late-Phase Consolidation of Opiate Reward Memories Requires Distinct Molecular and Temporal Mechanisms in the Amygdala-Prefrontal Cortical Pathway

**DOI:** 10.1371/journal.pone.0063612

**Published:** 2013-05-16

**Authors:** Shervin Gholizadeh, Ninglei Sun, Xavier De Jaeger, Melanie Bechard, Lique Coolen, Steven R. Laviolette

**Affiliations:** Department of Anatomy and Cell Biology, Department of Psychiatry, Schulich School of Medicine and Dentistry, University of Western Ontario, London, Ontario, Canada; Florida State University, United States of America

## Abstract

The consolidation of newly acquired memories involves the temporal transition from a recent, less stable trace to a more permanent consolidated form. Opiates possess potent rewarding effects and produce powerful associative memories. The activation of these memories is associated with opiate abuse relapse phenomena and the persistence of compulsive opiate dependence. However, the neuronal, molecular and temporal mechanisms by which associative opiate reward memories are consolidated are not currently understood. We report that the consolidation of associative opiate reward memories involves a temporal and molecular switch between the basolateral nucleus of the amygdala (BLA) (early consolidation phase) to the medial prefrontal cortex (mPFC) (late consolidation phase). We demonstrate at the molecular, behavioral and neuronal levels that the consolidation of a recently acquired opiate reward memory involves an extracellular signal-related kinase (ERK)-dependent phosphorylation process within the BLA. In contrast, later-stage consolidation of a newly acquired memory is dependent upon a calcium-calmodulin-dependent (CaMKII), ERK-independent, mechanism in the mPFC, over a 12 hr temporal gradient. In addition, using *in vivo* multi-unit neuronal recordings in the mPFC, we report that protein synthesis within the BLA modulates the consolidation of opiate-reward memory in neuronal mPFC sub-populations, via the same temporal dynamic.

## Introduction

Opiates are powerfully addictive drugs that create potent associative memories. Indeed, the persistence of opiate addiction is in large part due to the ability of opiate-related memories to trigger compulsive drug seeking and relapse, even years after abstinence. Memory formation and recall is a complex process involving initial acquisition, consolidation and reconsolidation phases, each linked to unique molecular and neuroanatomical mechanisms [1], 2. Importantly, emotionally salient memories undergo reorganization in a temporally dependent manner, from a recently acquired, less stable trace, to a longer-term, more permanent representation following a process of consolidation [3], [4], [5]. However, the temporal, neuroanatomical and molecular mechanisms that control the consolidation of recent vs. remote drug-related reward memories are not currently understood.

The basolateral amygdala (BLA) and prefrontal cortex (PFC) form an integrated neural circuit critical for the encoding and recall of emotional memory [6]–[11]. Neurons within the mPFC actively encode opiate-related reward memories and demonstrate associative increases in firing and bursting activity during the active recall of previously consolidated opiate reward memories [10]. Neurons within the BLA are required also for the acquisition of opiate reward and withdrawal aversion-related memories [8], [9]. Interestingly, µ-opiate receptor sensitive dopamine (DA) substrates within the ventral tegmental area (VTA), send projections directly to neuronal populations within the BLA [12], suggesting that opiate-related activation of DAergic VTA outputs may first target associative neuronal substrates within the BLA [9]. Furthermore, both the encoding and recall of reward and aversion-related associative memory involves functional interactions between the BLA and mPFC. We have reported recently that pharmacological inactivation of the BLA prior to the acquisition of associative opiate reward learning, leads to a disinhibition of mPFC neuronal population activity and a concomitant acceleration in the behavioral extinction of a previously acquired associative opiate reward memory. In contrast, BLA inactivation during extinction training causes a delay in extinction memory recall, demonstrating that BLA neuronal activity can strongly modulate the processing of associative opiate-related learning and memory [13]. Indeed, active input from the BLA to the mPFC is required for mPFC neuronal encoding of emotionally salient associative memories, including both fear-related and opiate-related learning [14], [15]. However, the potential role for functional interactions between the BLA and mPFC during the consolidation of reward-related memories is not currently understood.

Given the known functional relationships between the BLA and mPFC, we hypothesized that the consolidation of a newly acquired opiate-related reward memory would involve a temporally-dependent consolidation transfer within the BLA→mPFC pathway. Specifically, we hypothesized that early, recent opiate-memory consolidation would depend upon the BLA, whereas remote opiate-related reward memory consolidation would involve a delayed temporal transfer to an mPFC-dependent substrate. We report that intra-BLA or mPFC protein synthesis or ERK/CaMKII inhibition blocks the consolidation of acute opiate reward memory through a temporal gradient: recent opiate reward memory requires BLA-dependent consolidation whereas remote memory consolidation switches to an mPFC-dependent substrate. Examining protein expression levels of phosphorylated vs. total ERK 1/2 levels within the BLA and mPFC revealed a similar temporal dissociation during recent vs. remote opiate-reward related memory consolidation phases. Finally, micro-array recordings of mPFC neuronal populations *in vivo* demonstrated that intra-BLA protein synthesis inhibition prevented later-phase, mPFC-dependent consolidation of opiate reward memory when applied immediately post-learning, but not at 12 hrs after memory acquisition. These findings demonstrate that early vs. late-phase consolidation of newly acquired opiate-related reward memories occur via separate temporal and molecular mechanisms within the BLA→mPFC pathway.

## Results

### Consolidation of Opiate Reward Memory Shows Different Temporal Patterns in the BLA and mPFC

**Figure 1:**
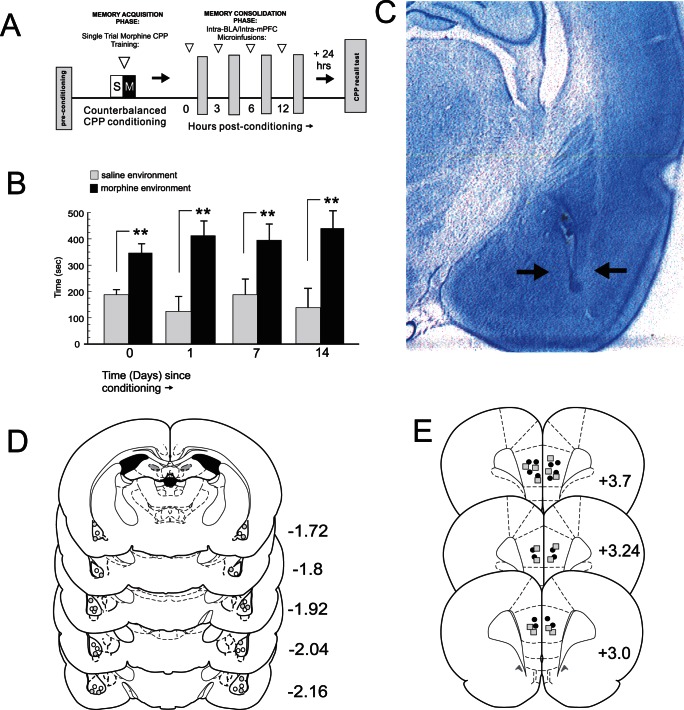
Experimental protocol, opiate reward memory CPP expression and intra-BLA histological analysis. ***A***, Schematic summary of single-trial, acute morphine reward memory CPP conditioning procedure. ***B***, Long-term stability of morphine single-trial CPP reward conditioning demonstrated by significant morphine-environment CPP up to 14 days post-conditioning. ***C*** **,** Microphotograph showing representative intra-BLA injector tip location (black arrows). ***D*** **,** Schematic summary of representative intra-BLA microinfusion sites for rats receiving intra-BLA ANI at 0 hrs (grey circles) or 12 hrs (white circles) post-conditioning. * = p<.05; ** = p<.01 for this and all subsequent figures. ***E,*** Schematic summary of representative intra-mPFC microinfusion sites for rats receiving intra-BLA ANI at 0 hrs (grey squares) or 12 hrs (black circles) post-conditioning. * = p<.05; ** = p<.01 for this and all subsequent figures.

**Figure 2:**
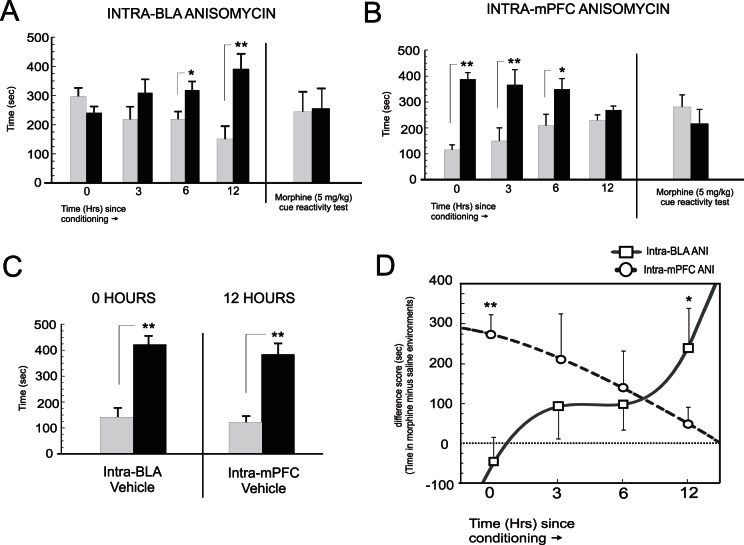
Effects of intra-BLA or mPFC anisomycin on temporal consolidation of acute opiate reward memory. *****A*****, Post-conditioning intra-BLA microinfusions of ANI completely block consolidation of opiate reward memory when administered at 0 and 3 hrs, but has no effect at 6 or 12 hrs. A Morphine cue-recall control test administered 24 hrs after the initial CPP test in the 0 hr group does not re-store the memory. ***B***, Post-conditioning intra-mPFC microinfusions of ANI completely block consolidation of opiate reward memory when administered at 12 hrs, but has no effect at time-points before this. A morphine cue-recall control test administered 24 hrs after the initial CPP test in the 12 hr group does not re-store the memory. ***C*** **,** Vehicle control groups for the effective time points of 0 hrs (intra-BLA) or 12 hrs (intra-mPFC) caused no block of morphine reward CPP memory consolidation. ***D,*** Temporally divergent effects of opiate reward memory consolidation interference presented as CPP difference scores (calculated as times in drug minus vehicle-paired environments).

Using a single-trial acute morphine reward conditioned place preference (CPP) paradigm (**Fig. 1A**) we first examined the stability and persistence of an acute, associative morphine reward memory, using a conditioning dose of morphine known to produce robust rewarding effects (5 mg/kg; i.p.) [10], [15], in previously opiate-naive rats (n = 6). Single trial morphine CPP conditioning produced a robust preference for morphine-paired environments that persisted for up to 14 days (**Fig. 1B**). One-way ANOVA of CPP scores revealed a significant main effect of treatment (saline vs. morphine; F_(7,39)_ = 6.17; p<.001) on times spent in morphine vs. saline-paired CPP test environments. Post-hoc analysis revealed significant preferences for morphine-paired environments when tested immediately post-conditioning (0 hrs), and at 1, 7 and 14 days post-conditioning (p′s<.01). Thus, a single morphine-environment pairing leads to the formation of a stable and persistent CPP memory lasting up to 2 weeks post-conditioning, similar to morphine conditioning memory dynamics following a longer, 8 day conditioning cycle [10], [16]. To determine the temporal dynamics of acute opiate reward memory consolidation, we targeted the consolidation phase of opiate reward memory encoding by performing bilateral microinfusions of the protein synthesis inhibitor anisomycin (ANI, 62.5 µg/0.5 µl; see methods) into the BLA (**Fig. 1C,D**) at 0, 3, 6 and 12 hrs post-conditioning. Histological analyses revealed intra-BLA micro-infusion locations to be localized within the anatomical boundaries of the BLA [17]. This dose of intra-BLA ANI has been reported previously to block fear memory reconsolidation when micro-infused *in vivo* [7]. Two-way ANOVA revealed a significant interaction between group and test session on times spent in saline vs. morphine-paired environments (F_(3,61)_ = 6.41; p<.01; **Fig. 2A**). Post-hoc analysis revealed that post-conditioning intra-BLA ANI blocked consolidation of morphine reward memory when administered at 0 (n = 8) and 3 hrs (n = 7) post-conditioning (p′s>.05). A smaller magnitude morphine CPP was present in the 6 hr group (p<.05; n = 8), but this preference was significantly lower (p<.05) than that observed for the 12 hr group (n = 8), who showed robust morphine CPP (p<.01). Thus, intra-BLA protein synthesis inhibition time-dependently blocks the consolidation of opiate reward memory over a 12 hr time course. To determine that CPP memory recall was entirely blocked and not simply a state-dependency phenomenon, we re-tested the intra-BLA 0 hr ANI group 24 hrs after the initial recall test, but gave a morphine-cue (5 mg/kg; i.p.) prior to CPP testing (see methods). Again, behavioral CPP expression was entirely absent during this morphine cued-recall test (t_(7)_ = 0.08; p>05).

Given our hypothesis that late-phase morphine reward memory consolidation occurs in the mPFC, we next performed bilateral ANI microinfusions into the mPFC following the same temporal protocol (**Fig. 1E**). Two-way ANOVA revealed a significant interaction between group and test session on times spent in saline vs. morphine-paired environments (F_(3,53)_ = 25.5; p<.0001; Fig. 2B). In direct contrast to the effects observed with intra-BLA ANI, post-hoc analysis revealed that post-conditioning intra-mPFC ANI blocked consolidation of morphine reward memory when administered at 12 (n = 7) hrs post-conditioning (p>.05). However, no block of morphine reward consolidation was observed for groups receiving intra-mPFC ANI at either 0 hr (n = 9; p<.01) 3 hr (n = 6, p<.05) or 6 hr time points (n = 6; p<.05). Again, to determine that CPP memory consolidation was behaviorally blocked and not simply a state-dependency phenomenon, we tested the intra-mPFC ANI 12 hr group, 24 hrs after the initial recall test, but gave a morphine-cue (5 mg/kg; i.p.) prior to CPP testing. Again, CPP memory was entirely absent during this cued-recall test (t_(5)_ = 0.66; p>05). Thus, intra-mPFC protein synthesis inhibition time-dependently blocks consolidation of opiate reward memory in the reverse temporal order to that observed with intra-BLA ANI administration, causing no blockade of recent opiate memory consolidation, but a complete blockade of late-phase memory consolidation at 12 hrs post-conditioning. To ensure that vehicle infusions were not blocking morphine CPP memory consolidation, separate control groups received either intra-BLA vehicle at the previously determined effective time-point of 0 hrs post-conditioning (n = 7) and an additional group received intra-mPFC vehicle at the previously determined effective time point of 12 hrs (n = 6; Fig. 2C). Analysis of CPP test scores revealed that neither intra-BLA vehicle at 0 hrs (t_(6)_ = 3.2; p<.01) nor intra-mPFC vehicle at 12 hrs (t_(5)_ = 4.4; p<.01) blocked consolidation of morphine reward CPP, with both groups demonstrating robust morphine (5 mg/kg; i.p.) CPP. We present a summary of time-dependent effects on morphine reward memory consolidation across intra-BLA and intra-mPFC groups in **Fig. 2D**, presented as difference scores. Analysis of difference scores revealed a significant interaction between group and time (F_(3,57)_ = 4.4, p<.01) with post-hoc analysis revealing significant divergence between CPP difference scores between intra-BLA vs. intra-mPFC groups at the 0 and 12 hr consolidation time points (p′s<.01 and <.05, respectively.

### Early vs. Late Phase Consolidation of Opiate Reward Memory Requires Intra-BLA and Intra-mPFC ERK and CaMKII Signaling

Given the temporal divergence in opiate-reward memory consolidation between the BLA and mPFC (**Fig. 2**), we next examined the underlying molecular substrates responsible for BLA vs. mPFC-mediated opiate-reward memory consolidation events. Two molecular substrates critical for synaptic plasticity and emotionally salient memory formation are the extracellular signal-regulated kinases (ERK) 1 and 2, and calcium calmodulin-dependent kinase II (CaMKII) 18–21. Testing our previously determined early vs. late-phase CPP memory consolidation time points (0 and 12 hrs), we pharmacologically targeted either ERK1/2 or CaMKII signalling within the BLA and mPFC by performing bilateral microinfusions of the CaMKII inhibitor KN-62 (0.05–0.5 µl/0.5 µl) or the ERK1/2 specific inhibitor U0126 (0.1–1.0 µg/0.5 µl), using dose ranges that have been reported previously to be behaviorally effective following *in vivo* intra-cranial microinfusions [22]–[24].

**Figure 3:**
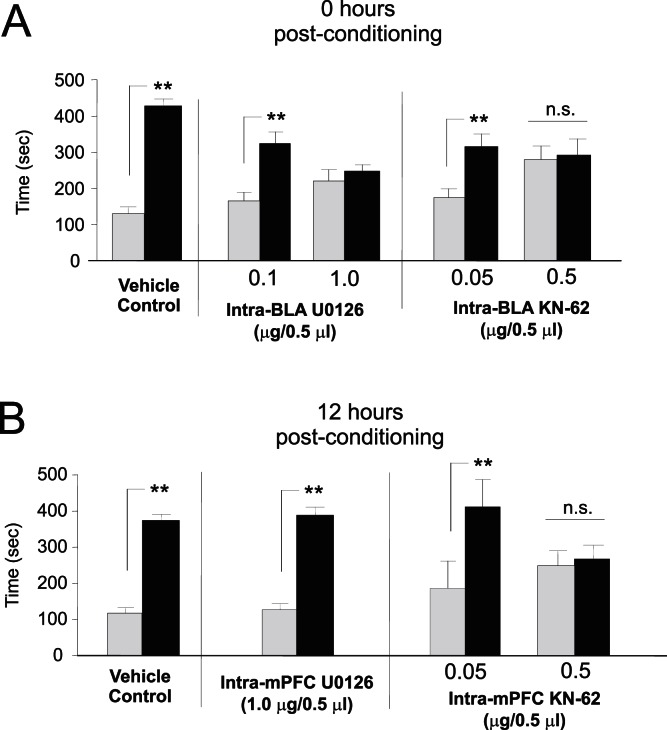
Roles of ERK and CaMKII signalling during recent and remote opiate memory consolidation. ***A*** **,** Relative to vehicle controls, both intra-BLA U0126 (0.1–1.0 µg/0.5 µl) and KN-62 (0.05–0.5 µg/0.5 µl), dose-dependently block opiate reward memory consolidation when administered at the 0 hr time point. ***B*** **,** Relative to vehicle controls, intra-mPFC KN-62, dose-dependently blocked opiate reward memory consolidation when administered at the 12 hr time point, while U0126 has no effect.

Analysis of morphine (5 mg/kg; i.p.) CPP scores tested 24 hrs after intra-BLA administration of U0126 (0.1–1.0 µg/0.5 µl) at 0 hrs post-conditioning, revealed that ERK inhibition within the BLA dose-dependently blocked consolidation of morphine reward memory. Two-way ANOVA revealed a significant effect of treatment (F_(2,45)_ = 12.58, p<.05) on times spent in morphine vs. saline-paired test environments. Post hoc analysis revealed that similar to vehicle controls, the group receiving a lower dose of U0126 (0.1 µg/0.5 µl; n = 6) demonstrated robust morphine CPP (p<.01). However, morphine CPP was completely blocked in the group receiving the higher dose of U0126 (1.0 µg/0.5 µl; n = 7; p>.05, **Fig. 3A** **, left side**). Similarly, analysis of CPP scores tested 24 hrs after intra-BLA administration of KN-62 (0.05–0.5 µg/0.5 µl) at 0 hrs post-conditioning revealed that CaMKII inhibition dose-dependently blocked consolidation of morphine reward memory. Two-way ANOVA revealed a significant effect of treatment (F_(2,43)_ = 9.68, p<.001) on times spent in morphine vs. saline-paired test environments. Post hoc analysis revealed that, relative to vehicle controls, the group receiving a lower dose of KN-62 (n = 7) demonstrated robust morphine CPP (p<.01). However, CPP was completely blocked in the group receiving the higher dose of KN-62 (n = 8; p>.05, **Fig. 3A** **, right side**).

We next administered the previously determined highest behaviorally effective dose of U0126 (1 µg/0.5 µl) into the mPFC at the 12 hr consolidation time point. Two-way ANOVA on scores recorded 24 hrs after intra-mPFC administration of U0126 (1.0 µg/0.5 µl) revealed a significant effect of treatment (morphine vs. saline) on times spent in morphine vs. saline environments (F_(1,27)_ = 92.1; p<.001) and post-hoc analysis revealed that both vehicle (n = 8) and U0126 groups (n = 7) demonstrated robust morphine CPP (p′s<.01; **Fig. 3B**, **left side**). For groups receiving intra-mPFC KN-62 (0.05–0.5 µg/0.5 µl) ANOVA revealed a significant effect of dose (F_(2,43)_ = 13.75, p<.001) on times spent in morphine vs. saline-paired test environments. Post hoc analysis revealed that whereas the group receiving vehicle (n = 8) or a lower dose of KN-62 (n = 6) demonstrated robust morphine CPP (p′s<.01), CPP was completely blocked in the group receiving the higher dose of KN-62 (n = 8; p>.05, **Fig. 3B** **, right side**). Thus, while blockade of either ERK or CaMKII signalling within the BLA at the 0 hr memory consolidation time point dose-dependently blocks morphine reward memory consolidation, late-phase memory consolidation within the mPFC was ERK-independent, suggesting a shift to an ERK-independent molecular memory substrate.

### Phosphorylated ERK Expression Levels are Differentially Regulated during Early vs. Late-phase Opiate Reward Memory Consolidation Phases in the BLA→mPFC Pathway

**Figure 4:**
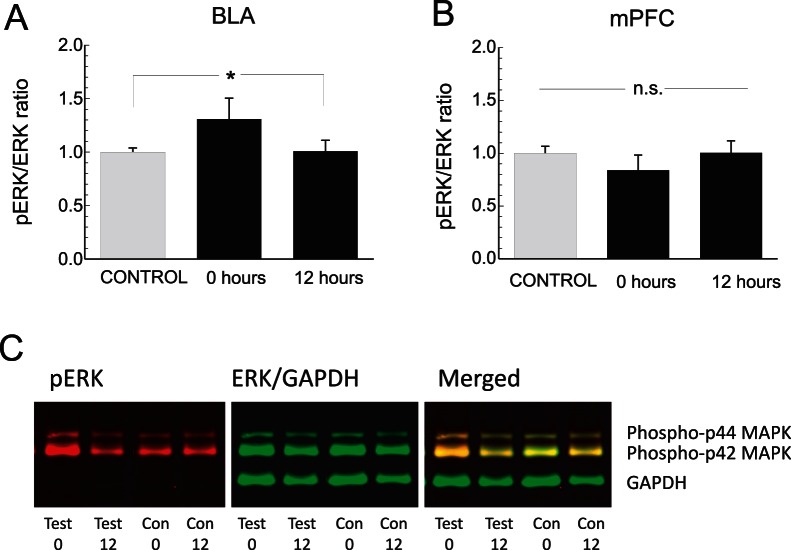
Analysis of pERK and ERK 1/2 protein expression levels in BLA and mPFC tissue during 0 and 12 hr post-conditioning memory consolidation time-points. ***A*** *,* Analysis of BLA tissue samples at 0 hr post-conditioning reveals significantly elevated pERK levels relative to total ERK levels, but not in vehicle control or 12 hr post-conditioning experimental groups. ***B*** *,* In contrast, no significant differences across groups are observed in the pERK/ERK ratio analysed in mPFC tissue samples. *C*, Representative Western Blot samples showing fluorescent imaging of pERK and ERK/GADPH bands across experimental groups at 0 and 12 hr post-conditioning time-points.

Our behavioral findings demonstrated that BLA-dependent consolidation of early opiate reward memory is ERK-dependent whereas later stage consolidation is independent of ERK signaling in the mPFC (**Fig. 3A**). In contrast, we observed no functional or temporal differences between the effects of intra-BLA or mPFC CaMKII blockade. Given these differential behavioral effects following ERK or CaMKII inhibition, we next examined and compared protein expression levels of ERK1/2 and phosphorylated ERK1/2 (p-ERK) as a function of post-conditioning memory consolidation phases in the BLA→mPFC pathway (see methods). (**Fig. 4**). First, comparing the expression ratio of pERK to ERK within the BLA revealed a significant increase in ratio magnitude specifically in BLA tissue analyzed at 0 hr post-conditioning (n = 5), relative to vehicle conditioning controls (n = 10) or in tissue analyzed at the 12 hr time-point (n = 5). One-way ANOVA revealed a significant effect of group on relative pERK/ERK BLA expression levels (F_(2,29)_ = 6.1; p<.01), with post-hoc analysis revealing a significantly elevated ratio in the 0 hr experimental group, relative to vehicle controls or the 12 hr post-conditioning group (**Fig. 4A**; p<.05). In contrast, comparing pERK/ERK ratio levels in the mPFC revealed no differences across control (n = 10), 0 (n = 5) or 12 hr (n = 5) experimental groups (F_(2, 29)_ = 2.61; p>.05; **Fig. 4B**). Expression levels of GAPDH and of ERK (analyzed as ratio over GAPDH levels) did not significantly differ between groups (data not shown). In **Fig. 4C**, representative Western blot data from the BLA is presented, showing elevated relative pERK levels in the 0 hr test group, compared to vehicle control or 12 hr test groups.

### Protein Synthesis Inhibition in the BLA Modulates Associative Neuronal Encoding of Opiate Reward Memory in the mPFC

**Figure 5:**
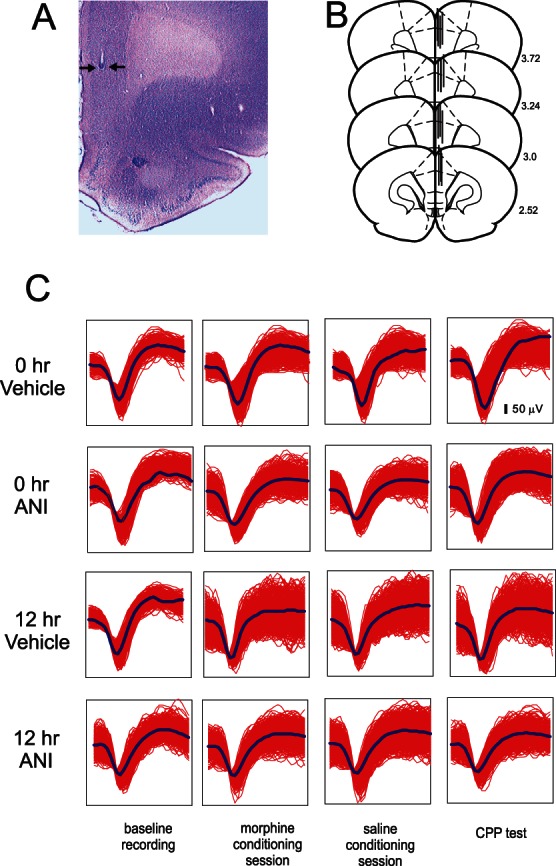
Intra-mPFC microwire recording locations, waveform analysis and effects of intra-BLA ANI on neuronal encoding of opiate reward memory. ***A***, Microphotograph showing a representative intra-mPFC recording wire placement (black arrows). ***B***, Schematic representation of sample intra-mPFC microwire recording location, demonstrating rostral-caudal distribution within the mPFC. ***C***, Sample mPFC neuronal unit waveforms from single channels, recorded over 3 days (baseline, conditioning day, CPP recall test) from four experimental groups receiving either intra-BLA vehicle ANI or vehicle at 0 or 12 hrs post-conditioning ***D***.

**Figure 6:**
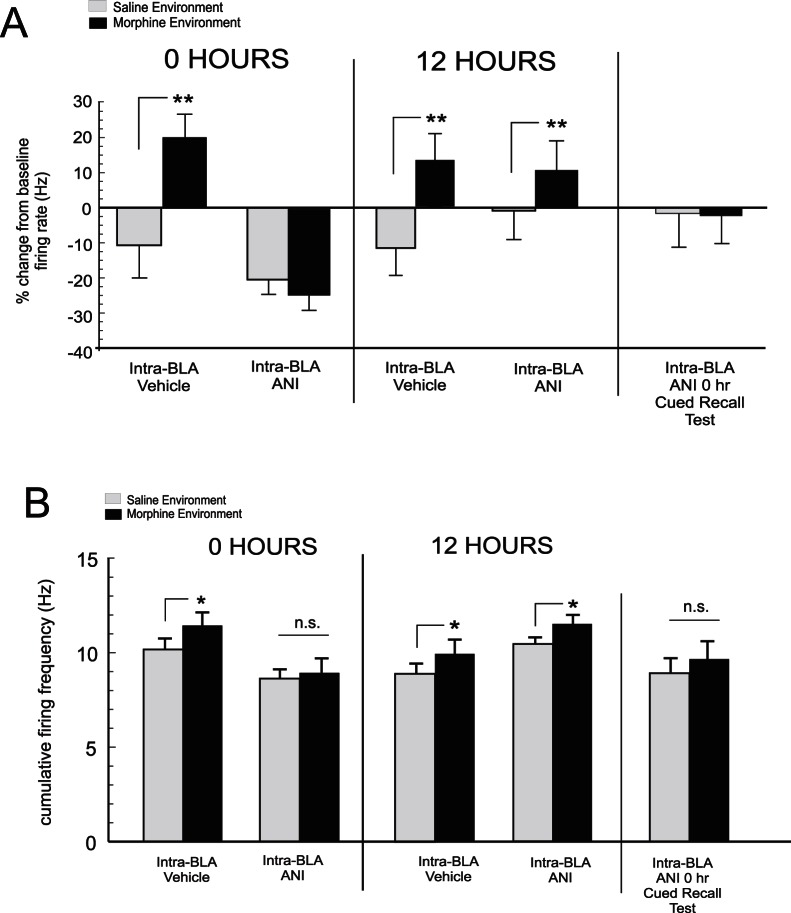
Effects of acute (0 hr) intra-BLA protein synthesis inhibition on mPFC neuronal associative morphine responses during CPP testing. *A*, When administered at 0 hrs post-conditioning, intra-BLA ANI blocks mPFC neuronal encoding of associative opiate reward memory, demonstrated by a lack of associative firing activity increases during exposure to morphine environments during CPP testing or during a morphine cued recall CPP test. In contrast, vehicle controls at 0 or 12 hrs or intra-BLA ANI administration at 12 hrs, does not block mPFC neuronal encoding of opiate reward memory, demonstrated by robust mPFC neuronal firing activity during CPP exposure to morphine-paired environments. B, Cumulative mPFC neuronal firing frequency rates are presented for the same experimental groups shown in panel A. Cumulative neuronal firing frequency demonstrates morphine-environment specific associative increases in firing rates relative to saline-paired environments in groups receiving intra-BLA vehicle at 0 or 12 hrs post-conditioning or in rats receiving intra-BLA anisomycin at 12 hrs post-conditioning. However, this associative increase in firing activity is absent in rats receiving intra-BLA anisomycin at 0 hrs post-conditioning.

Neuronal subpopulations within the mPFC are involved in the encoding and expression of opiate-related reward memory [10], [13]. Having established the temporal dynamics between the BLA and mPFC during opiate reward memory consolidation, we next sought to determine how memory consolidation disruption directly within the BLA may modulate mPFC neuronal encoding of associative opiate reward memory. Using 8 channel micro-array *in vivo* recordings in the mPFC (see methods, **Fig. 5A**–**B**) during our above described single-trial morphine conditioning assay, we next examined how intra-BLA ANI microinfusions, targeting the previously established 0 and 12 hr memory consolidation time-points, might modulate later associative mPFC neuronal activity during CPP recall testing (**Fig. 1A**). As described previously, mPFC neurons show relatively consistent waveform stability when recorded over multiple conditioning days [10], [13]. Nevertheless, given the long-term nature of these recording sessions, it is important to note that distinct neuronal units may be differentially recorded across days (see methods; Fig. 5C). Two-way ANOVA Comparing mPFC neuronal activity levels across saline or morphine-environment exposure during CPP recall testing revealed a significant effect of group on mPFC neuronal frequency levels (F_(3,151)_ = 11.41; p<.0001; **Fig. 6**). Post hoc analysis revealed that in rats receiving intra-BLA vehicle at 0 hrs post-conditioning (n = 7; n = 34 neuronal units); associative mPFC neuronal activity was significantly increased in morphine vs. saline-paired environments, relative to baseline activity levels (p<.01). However, in rats receiving intra-BLA ANI at 0 hrs post-conditioning (n = 7, n = 36 neuronal units), no associative increase in mPFC neuronal activity was observed during CPP testing comparing exposures to either saline or morphine-paired environments (p′s>.05). Thus, similar to prior behavioral effects (**Fig. 2A**), intra-BLA ANI administration at 0 hrs, blocked associative mPFC neuronal activity during recall testing (**Fig. 6A**). In contrast, for rats receiving intra-BLA vehicle (n = 7; n = 29 neuronal units) or ANI administration (n = 7; n = 31 neuronal units) at 12 hrs post-conditioning, mPFC neurons demonstrated significant associative increases in firing activity specifically during exposure to morphine-environments during CPP testing (**Fig. 6A**), demonstrating the establishment of remote morphine reward memory in mPFC neuronal populations, consistent with prior behavioral results (**Fig. 2B**). Furthermore, post-hoc analysis revealed no significant differences between morphine-environment mPFC neuronal firing rates across rats receiving intra-BLA ANI at 0 hrs post-conditioning vs. rats receiving intra-BLA vehicle or ANI at 12 hrs post-conditioning (p′s>.05). Again, to ensure that morphine reward memory was absent in the BLA 0 hr group, a subsequent CPP test was performed 24 hrs later, giving rats a morphine cue (5mg/kg; i.p.) immediately prior to testing. Analysis of mPFC neuronal activity during exposure to morphine vs. saline environments during this control test revealed no significant differences from baseline activity levels across morphine and saline environments (t_(71)_ = 0.78; p>.05; **Fig. 6A** **, far right side**). In **Fig. 6B**, cumulative firing frequency activity recorded during exposure to saline or morphine paired environments during CPP recall testing is presented. Cumulative firing activity was calculated as the total average firing rate, per unit, comparing each experimental group across conditions. Analysis of cumulative firing frequency across experimental groups revealed a significant effect of group (F_(7,167)_ = 2.3; p<.05) on mPFC neuronal firing frequency rates recording during CPP tests. Post-hoc analysis revealed that firing rates during exposure to morphine-paired environments were significantly elevated relative to saline-environmental exposure in rats receiving intra-BLA vehicle at 0 hrs and in rats receiving intra-BLA vehicle or ANI at 12 hrs post-conditioning (p′s<.05). However, in rats receiving intra-BLA ANI at 0 hrs post-conditioning or a morphine-cue (5mg/kg; i.p.) prior to CPP testing, no significant difference in firing frequency across morphine vs. saline environments was observed during CPP testing (**Fig. 6B**).

**Figure 7:**
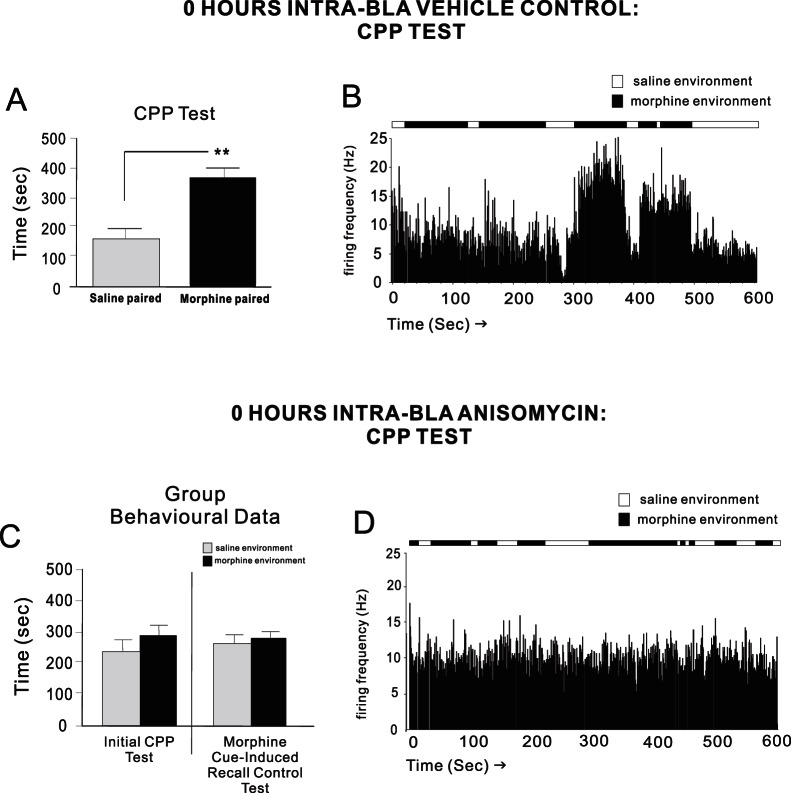
Effects of acute (0 hr) intra-BLA protein synthesis inhibition on behavioral CPP expression and mPFC neuronal activity. ***A*** *,* Group data showing robust behavioral morphine CPP from rats receiving intra-BLA vehicle at 0 hrs post-conditioning. ***B*** *,* A sample rastergram from a single rat, showing typical mPFC neuronal associative responding across saline vs. morphine-paired environments during real-time CPP testing. ***C*** *,* Group data showing blockade of behavioral morphine CPP expression from rats receiving intra-BLA anisomycin at 0 hrs post-conditioning. ***D*** *,* Sample rastergram from a single rat, showing mPFC neuronal responding during real-time CPP testing, across saline or morphine-environmental exposure. Associative neuronal increases during morphine environmental exposure is absent, consistent with a lack of behavioral CPP expression.

**Figure 8:**
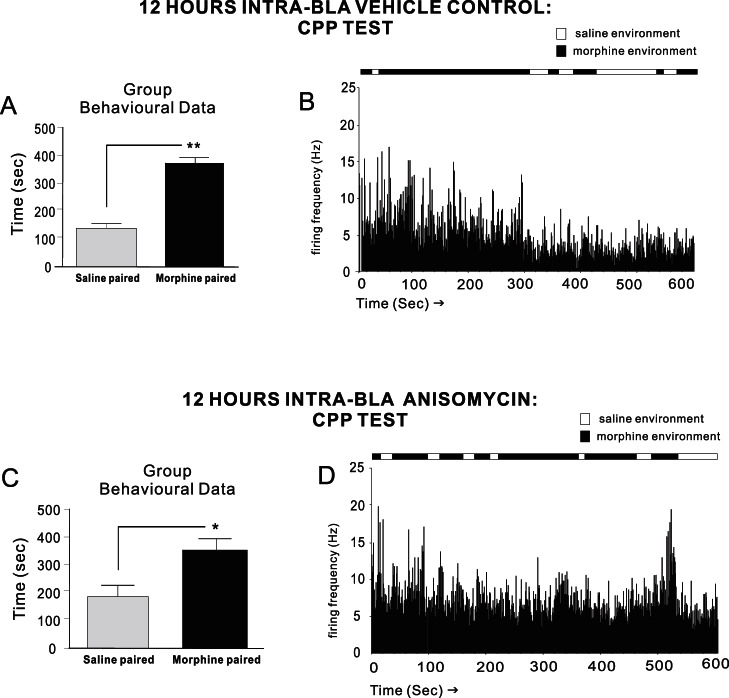
Effects of later (12 hr) intra-BLA protein synthesis inhibition on behavioral and mPFC neuronal opiate memory consolidation. Group CPP data showing that intra-BLA vehicle ***A*** *,* or anisomycin administration (***C***) at 12 hrs post-conditioning does not block subsequent behavioral recall of consolidated opiate reward memory. In addition, mPFC neuronal populations show robust, associative firing rate increases in response to morphine environment exposures during real-time CPP test recordings (panels ***B*** *,* ***D***; raster samples taken from single rats showing mPFC neuronal firing recorded in real-time across saline or morphine-environmental exposure).

For experimental groups used for intra-mPFC neuronal recording experiments, two-way ANOVA on CPP test scores revealed a significant effect of treatment on times spent in morphine vs. saline-paired environments (F_(1,55)_ = 25.1; p<.001). Post-hoc analysis revealed that rats receiving intra-BLA vehicle (n = 7) at 0 hrs post-conditioning displayed robust morphine CPP during testing (p<.01; **Fig. 7A**). A representative CPP recording rastergram from one of these rats showing associative mPFC neuronal firing increases during saline vs. morphine environment exposure, in real time, is presented in **Fig. 7B**. In direct contrast, intra-BLA ANI at 0 hrs (n = 7) completely blocked morphine CPP memory consolidation both during the initial recall test (p>.05) or when tested during morphine cue (5 mg/kg; i.p.) exposure (t_(6)_ = 0.08; p>.05; **Fig. 7C**). A sample rastergram from a rat receiving intra-BLA ANI at 0 hrs post-conditioning is presented in **Fig. 7D**, demonstrating the complete absence of associative mPFC neuronal activity during exposure to morphine environments during real time CPP testing. Finally, for experimental groups receiving intra-BLA vehicle or ANI (n = 7) at 12 hrs post-conditioning (n = 7), robust behavioral morphine CPP was observed (p′s<.01 and <.05, respectively. **Fig. 8A,C**), concomitant with the presence of associative mPFC neuronal firing activity specifically during exposure to morphine environments during CPP testing (**Fig. 8B,D**). Thus, blockade of intra-BLA protein synthesis at the previously established time points (0 and 12 hrs) for recent vs. remote opiate reward memory consolidation, specifically prevented the neuronal encoding and subsequent neuronal expression of associative opiate reward memory, when early phase (0 hr) memory consolidation was targeted in the BLA.

## Discussion

Memory formation has been hypothesized to involve distinct temporal phases of encoding and consolidation. McGaugh [3] defined short-term memory, lasting minutes to hours, and a longer term memory process that consolidates slowly, representing a more permanent memory trace. Previous research has examined and dissociated the underlying neuronal and molecular substrates involved in recent vs. remote memory formation, particularly in the context of fear-related memories [2], [21]. This evidence has suggested functional relationships between the hippocampus and cortex as crucial for the transfer and consolidation of recent vs. remote associative memory [2]. In addition, re-activated fear-related memories undergo a labile period of reconsolidation during which time they are vulnerable to interference [7]. Interestingly, targeted protein synthesis inhibition, at least within the BLA, does not appear to disrupt the later recall of opiate-related reward memories but does block cocaine-associated reward memories [19], [25]. Nevertheless, the temporal and neuroanatomical characteristics of acute opiate reward memory consolidation have not previously been characterized.

In terms of drug addiction-related reward memory, opiate-related memories are correlated with associative activity in mPFC neuronal circuits [10], [26]. We have reported previously that neurons within the mPFC show morphine-related associative increases in firing and bursting activity both during the initial acquisition and during the behavioral recall of these memories [10], [13]. Furthermore, these associative neuronal responses in the mPFC are observable after a 24 hr behavioral conditioning time-frame [10], consistent with the present results demonstrating that the consolidation of a newly acquired associative opiate reward memory switches to an mPFC-dependent substrate within 12 hrs. Indeed, the recall of opiate-related memory in human addicts and rodents is correlated with the activation of the mPFC, consistent with the notion that long-term opiate addiction related memory is encoded in mPFC circuits [27]–[29]. In the present study, we found that protein synthesis inhibition within the BLA during early phase memory consolidation, blocked the subsequent neuronal expression of morphine-related associative firing increases when tested in the CPP paradigm. This effect on mPFC neuronal read-out was consistent with behavioral results showing that intra-BLA protein synthesis inhibition blocked associative opiate reward memory expression when consolidation was targeted at 0 hrs post-conditioning, but not at 12 hrs post-conditioning. While future studies are required to precisely examine how neuronal populations within the BLA encode opiate related memories over this temporal framework and beyond, one possibility is that targeting a BLA-dependent consolidation process during early consolidation, prevented the subsequent transfer of morphine-related associative memory to the mPFC for later phase consolidation. In contrast, an alternate possibility is that intra-BLA protein synthesis inhibition may interfere with neuronal activity parameters within mPFC neuronal populations, leading to interference with neuronal activity dynamics within the mPFC. Indeed, we have recently reported that intra-BLA cannabinoid receptor modulation can strongly modulate spontaneous neuronal activity within mPFC neuronal sub-populations in the context of fear-related memory encoding, demonstrating that modulation of signaling within the BLA can functionally influence spontaneous neuronal activity within the mPFC [11]. In addition, it is possible that mPFC neuronal activity patterns may represent a form of associative place memory, independent of the motivational properties of morphine *per se*.

The functional relationship between the BLA and mPFC during the processing of emotionally salient associative memory is complex. Thus, bi-directional efferent and afferent connectivity between these neural regions can modulate neuronal activity patterns within both the BLA and mPFC [14], [30]. However, considerable evidence suggests that the direction of emotional memory formation within this circuit involves ascending input from the BLA to neurons in the mPFC [11], [14], [15]. Synaptic plasticity in the form of long-term potentiation along the BLA→mPFC pathway is associated both with the experience of acute emotionally salient events [14], [31] and with the formation, recall and extinction of associative fear memories [11], [14]. The present findings suggest several potential mechanisms for the temporal and neuroanatomical consolidation substrates responsible for opiate-related reward memory. One possibility is that the BLA serves as a first-order memory integration centre for early phase consolidation of opiate reward memory. Following which, an mPFC-dependent mechanism becomes responsible for longer-term consolidation of the associative memory over specific temporal constraints. Alternatively, given the critical functional and anatomical connectivity between the BLA and mPFC, memory substrates within the BLA may modulate a longer term consolidation process within the mPFC, via functional inputs to the mPFC. Such a relationship may be suggested by recent findings that BLA inactivation prior to the ‘acquisition’ phase of opiate-reward memory leads to a destabilized and accelerated behavioral extinction of opiate-reward memory [13]. While future studies are required to resolve these issues, the present findings demonstrate an important distinction between the BLA and mPFC in terms of the temporal dynamics of reward-related associative memory consolidation.

In the present study, we found that the consolidation of opiate reward memory depends upon both ERK and CaMKII signaling within the BLA→mPFC pathway, two molecules that are involved critically in the processing of short and long-term associative memory. For example, CaMKII and ERK blockade prevents both induction and stability of long-term potentiation within the hippocampus [32]. Inhibition of ERK signalling has been reported to disrupt psychostimulant reward reconsolidation memory [18], [19] and ERK signalling has been implicated in the mediation of short-term associative memory formation within the hippocampus [33]. In the present study, we found elevated intra-BLA pERK levels specifically at an early phase memory consolidation time point (0 hrs). This elevation in pERK levels was not observed in the mPFC, consistent with our behavioral findings demonstrating that later phase opiate reward memory consolidation can be mediated through an ERK-independent substrate.

In addition to ERK signaling, we found that intra-BLA and intra-PFC CaMKII signalling was required for the behavioral consolidation of early and late-phase opiate reward memory whereas the consolidation of late-phase opiate reward memory was ERK-independent. While future studies are required to examine the underlying functional mechanisms related to ERK 1/2 signalling within the BLA→mPFC circuit, the present results suggest a functional role for ERK signaling during recent opiate memory consolidation specifically within the BLA. Furthermore, the present findings with ERK signaling are consistent with previous reports demonstrating that psychostimulant drugs in general are capable of causing acute activation of ERK1/2 signaling [19], [34]. Interestingly, Languille et al. [35] reported that intra-amygdala ERK phosphorylation was critical for post-acquisition fear memory consolidation or post-reactivation consolidation when interrupted 1–6 hrs post-conditioning. However, it is important to note that intra-BLA ERK inhibition was not tested in the present study at later time-points. Hence, it is possible that ERK may still be involved in opiate associative memory consolidation at times beyond 0 hrs. Indeed, previous evidence has demonstrated the involvement of ERK signaling during later-phase associative memory processing. Thus, Schafe et al. [36], using a single-trial fear-conditioning assay, reported that intra-BLA ERK inhibition with U0126 blocked the acquisition of long-term, but not short-term associative fear memories, while intra-BLA ERK phosphorylation peaked at 60 min post-conditioning. While the present study examined only the post-conditioning consolidation phase of reward-related memory, this may suggest that intra-BLA ERK signaling may require different temporal dynamics during acquisition vs. consolidation phases of emotionally salient associative memory processing. In addition, the present study examined only ERK and CaMKII signalling mechanisms within the BLA→mPFC circuit. Future studies are required to examine other potential molecular substrates that may sub-serve opiate-related associative memory formation during early vs. later consolidation phases.

The BLA itself is not involved in processing the primary rewarding properties of opiates [37]. Rather, considerable evidence implicates the ventral tegmental area (VTA) as a critical neural substrate for primary opiate-reward signalling as direct opiate administration produces potent reinforcing effects in the VTA [16], [38]. Nevertheless, the BLA receives extensive dopaminergic (DAergic) inputs from the VTA that have been shown to modulate neuronal plasticity mechanisms within amygdalar neuronal populations [39]. Furthermore, DAergic transmission within the BLA is required for the acquisition of opiate-related reward memories [9]. Functionally, µ-opiate responsive DAergic outputs from the VTA preferentially target BLA neurons [12], suggesting that DAergic signalling in response to opiate exposure modulates neuronal activity within the BLA, consistent with previous reports demonstrating strong DAergic modulation of BLA neuronal populations [39], [40]. In contrast, VTA DAergic projections to the nucleus accumbens (NAc) have been reported to respond to kappa-receptor sensitive neuronal populations within the VTA [12]. Nevertheless, the VTA also sends DAergic projections directly to the mPFC [41], [42]. In addition, glutamatergic hypofunction directly in the mPFC potentiates opiate-related reward memory through DA-dependent substrates [15], [43] and modulation of mPFC neuronal activity via ascending BLA inputs is dependent upon DAergic signaling [44]. However, in these cases, DAergic modulation of emotionally salient memory is dependent upon functional BLA inputs, consistent with the current findings demonstrating that the BLA represents a first-order neural substrate for the processing of opiate-related associative memory.

Given that BLA neurons integrate emotionally salient associative memory via DAergic and extrinsic cortical sensory inputs [45], our evidence suggests that the BLA may serve as a first-order opiate-reward memory integration center for the consolidation of recent drug-related memory formation. This is consistent with previous research demonstrating an ascending, functional link between the BLA and mPFC during the processing of emotionally salient associative memories, both at the level of the single mPFC neuron [14], [46] and in terms of plasticity mechanisms associated with memory formation, such as long-term potentiation [11], [31]. While future studies are required to determine the long-term retention parameters of opiate-related memories within the mPFC, the present results demonstrate a temporally and neuroanatomically-dependent transfer of an associative opiate reward memory, from sub-cortical to cortical memory consolidation substrates.

## Methods

### Ethics Statement

All procedures were approved by the Animal Care and Use Committee of the University of Western Ontario and conformed to Canadian Council on Animal Care guidelines involving vertebrate animals in research.

#### Animals and surgery

Male Sprague–Dawley rats (350–400 gm) were anesthetized with a ketamine (80 mg/ml) xylazine (6 mg/ml) mixture and placed into a stereotaxic device. Incisions were made in the scalp to expose the skull, and burr holes were drilled and the dura was removed overlying the mPFC and BLA regions. For bilateral intra-BLA/mPFC guide cannulae implantation, stainless steel guide cannulae (22 gauge; Plastics One) were implanted using the following stereotaxic coordinates (in mm). For the BLA: from bregma AP −3.0, L ±5.0; from the dural surface, V −7.4. For the mPFC (15° angle: from bregma AP +2.9, L ±1.9; from the dural surface, V −3.0. For mPFC neuronal recording studies, 8-channel microwire arrays (Tucker-Davis Inc.) were slowly lowered unilaterally into the mPFC region using the following stereotaxic coordinates (16) from bregma (mm): anteroposterior (AP) = +2.9; lateral (L) = −0.7; ventral(V) = −3.4 from the dural surface. Following behavioral experiments, rats were deeply anesthetized with an overdose of Sodium Pentobarbitol (Euthanyl, 270 mg/kg; i.p.) and intra-cardially perfused with isotonic saline, followed by 10% formalin. The brains were removed and cut in 40 µm sections and stained with Cresyl Violet for microscopic analysis.

#### Drugs and injection procedures

Morphine (morphine hydrochloride, MacFarlane Smith), was dissolved in physiological saline (pH adjusted to 7.4). Anisomycin (Tocris, 62.5 µg/0.5 µl/side) was dissolved in 0.1 ml of 1 M HCl with pH adjusted to 7.2 by titration of 10 M NaOH. U0126 and KN-62 were dissolved in a 1∶1 vehicle solution consisting of DMSO and physiological saline (pH adjusted to 7.4). Bilateral intra-BLA or mPFC microinjections of ANI, KN-62, U0126 or their respective vehicles (0.5 µl volume per infusion) were performed over 1 min via plastic tubing connected to a 1 µl Hamilton micro-syringe. Injectors were then left in place for an additional 1 min to ensure adequate diffusion from the injector tip. Rats received intra-BLA/mPFC microinfusions after both saline and morphine conditioning sessions, thus, any potential non-specific effects of drug treatment are counterbalanced across conditioning environments.

#### Place conditioning procedure

Our conditioned place preference procedure is schematically described in **Fig. 1A**. Briefly, fully counterbalanced place conditioning took place in one of two distinct environments that differed in color, smell and texture. These conditioning environments are motivationally balanced such that rats show no initial preference for either environment before conditioning [16]. To further ensure that no systematic bias for either conditioning environment exists, we perform a post-test analysis, comparing total times spent in either conditioning environment during the CPP test, to determine that no systematic bias towards either conditioning environment exists within any particular experimental group. All conditioning and CPP testing was performed in the light phase. For CPP conditioning, rats were pre-conditioned via exposure to a neutral, grey environment distinct from saline or morphine conditioning boxes. On the next day, rats received a systemic injection of saline vehicle and placed into the assigned control conditioning environment for 30 min. Two hrs later, rats received an injection of morphine (5 mg/kg; i.p.) and placed in the alternate conditioning environment for 30 min. The conditioning environments were randomly assigned in a counterbalanced order, as described previously [10], [16]. In order to target specific time points during the memory consolidation phase, separate experimental groups received bilateral microinfusions of either ANI, KN-62, U0126 or their respective vehicles at 0, 3, 6 or 12 hrs following the morphine conditioning session. Rats were then returned to the home cage and were given a CPP recall test 24 hrs following the micro-infusions. For the CPP test phase, times spent in saline vs. morphine-paired environments were digitally recorded over a 10 min time course.

#### Multi-Unit electrophysiological recordings and analysis

Intra-mPFC recording procedures were identical to those previously described [10], [13]. Briefly, eight-channel microwire arrays (model MW8, Tucker-Davis Inc.) were used to record neuronal activity within the mPFC. Microwire arrays consisted of 8 wires arranged in 2 rows, separated by 500 microns and the anterior-posterior wire placements separated by 50 microns each. Microwire probes were connected to an RA-16PA pre-amplifier and then sent to a Pentusa Base Station (model RX5, Tucker-Davis Inc.). Neuronal spike signals were sampled at 25 kHz/channel with filter settings of 100 Hz (high pass) and 5 kHz (low pass) and were then sent to a window discriminator/amplifier. Neuronal activity data were simultaneously collected and monitored on-line using spike sorting software (Open Ex, Tucker-Davis Inc) and stored for off-line analysis. Spike waveforms were sorted and analyzed using K-means analysis software (OpenSort, TDT) offline. Subsequent electrophysiological analysis was performed with NeuroExplorer (NEX Technology), wherein ISI intervals are constructed for each isolated unit and compared across experimental sessions. Baseline activity recordings were performed prior to the beginning of each behavioral training session within the home cage for 5 min. We then compared baseline mPFC neuronal activity states across experimental recording sessions, within each experimental phase (pre-conditioning, acquisition, and recall). Our recording procedure and waveform analysis software links our online behavioral analysis with a continuous recording output from each array channel over the course of the 10 minute CPP test. For post-test analysis, isolated neuronal units were averaged across channels for each individual rat, and the summed activity recorded in either the saline or morphine-paired environments was calculated for each individual rat, following which group analysis was performed across the experimental groups to determine average mPFC neuronal firing rates in either the saline or morphine-paired environments.

#### Protein expression analysis

To examine protein expression levels of total and pERK during the consolidation phase of morphine reward CPP memory processing, separate experimental groups of rats (n = 5 per group) received the above described single-trial morphine (5 mg/kg; i.p.) conditioning procedure and were then sacrificed at either 0 or 12 hrs from the final conditioning session (see above). Brains were rapidly removed, frozen on dry ice, and stored at −80°C until processing. The BLA and PFC (including prelimbic, infralimbic, and anterior cingulate areas) were microdissected and homogenized mechanically in RIPA buffer [50 nM Tris-HCl; 150 mM NaCl; 1% Nonidet P 40; 0.1% sodium dodecyl sulphate (SDS); 0.5% sodium deoxycholate] supplemented with a protease inhibitor cocktail tablet (Roche USA). The homogenized mixture was centrifuged at 12,000 rpm for 20 min at 4°C and supernatant collected. Protein concentrations were determined using a BCA assay (ThermoFisher Scientific, Waltham, MA) and a NanoDrop ND-1000 spectrophotometer (ThermoFisher Scientific). Protein samples (10 µg) were boiled at 96°C for 4 minutes using an Accublock Digital Dry Bath (Labnet International, Edison, NJ) and loaded on a 10% polyacrylamide gel and separated under recuing conditions using a Mini Trans-Blot Cell system (Bio-Rad Laboratories Ltd.) and Tris-Glycine-SDS running buffer [25 mM Tris, 192 mM Glycine, 0.1% SDS (pH 8.3)]. Precision Plus protein All Blue standards (Bio-Rad Laboratories Ltd.) were used as molecular weight markers. Following separation, proteins were transferred to Millipore Immobilon-FL polyvinylidene difluoride membranes (PVDF; Millipore, Billerica, MA, USA) using the Trans-Blot Cell wet blotting system (Bio-Rad Laboratories Ltd.) for immunoblotting. The protein transfer from gel to membrane occurred in transfer buffer (20% methanol and 0.037% SDS in Tris-Glycine [25 mM Tris, 192 mM Glycine (pH 8.3)) (Bio-Rad Laboratories) at 82 V for 1 hr at RT. All samples were run in triplicate, and balanced across groups and individual gels. Next, membranes were incubated in a 2∶3 solution of Odyssey Blocking Buffer (LI-COR Biosciences, Lincoln, NE) and Tris-Buffered Saline (TBS; 50 mM Tris and 150 mM NaCl (pH8.0) for 1 hr under gentle agitation at room temperature. Membranes were then simultaneously incubated for 16 hrs at 4°C under gentle agitation with rabbit anti-phospho ERK (1∶4,000 phsopho-p44/p42, Cell Signaling Technology, Danvers, MA), mouse anti-ERK1/2 (1∶800, Santa Cruz Biotechnology, Santa Cruz, CA) and mouse anti-GAPDH (1∶200,000; MAB374; Millipore, Temecula, CA). Next, membranes were incubated for 1 hr in Alexa-680-cojugated goat anti-rabbit or donkey anti-goat (1∶5,000, Invitrogen, Carlsbad, CA) and IR Dye 800 CW-conjugated goat anti-mouse (1∶10,000; L-COR Biosciences). All antibodies were diluted in a 2∶3 mix of Odyssey Blocking Buffer with TBS-T (TBS +0.05% Tween-20 (pH8.0)). Bands of fluorescent immunoreactivity were visualized and images captured using an Odyssey 2.1 scanner (LI-COR Biosciences). For each protein sample, fluorescence intensity levels for each band were determined using the Odyssey software and averages were calculated for each animal. Ratios of ERK1/2/GAPDH, and pERK/ERK1/2 were determined for each protein sample and averages calculated for each animal. Mean ratios for the zero and twelve hr control groups were compared and found to be equal. Ratios for each animal in the experimental groups were expressed as a fold change over the mean of the combined zero and twelve hr control group and compared between groups using ANOVA.

#### Data analysis

All data are expressed as mean ± S.E.M and were analyzed with one or two-way analysis of variance (ANOVA) or student’s t-tests. Post-hoc analyses were performed with Bonferroni-Dunn tests or Fisher’s LSD tests where appropriate.
